# Effects of amniotic fluid on human keratinocyte gene expression: Implications for wound healing

**DOI:** 10.1111/exd.14515

**Published:** 2022-01-15

**Authors:** Erika Nyman, Elvira Lindholm, Jonathan Rakar, Johan P. E. Junker, Gunnar Kratz

**Affiliations:** ^1^ Department of Biomedical and Clinical Sciences Linköping University Linköping Sweden; ^2^ Department of Hand Surgery, Plastic Surgery and Burns Department of Biomedical and Clinical Sciences Linköping University Hospital Linköping Sweden; ^3^ Centre for Disaster Medicine and Traumatology Linköping University Hospital Linköping Sweden

**Keywords:** fetal wound healing, human skin cells, in vitro, microarray, PCR

## Abstract

Cutaneous wounds can lead to huge suffering for patients. Early fetal wounds have the capacity to regenerate without scar formation. Amniotic fluid (AF), containing hyaluronic acid (HA), may contribute to this regenerative environment. We aimed to analyse changes in gene expression when human keratinocytes are exposed to AF or HA. Human keratinocytes were cultured to subconfluence, starved for 12 h and then randomised to be maintained in (1) Dulbecco's modified Eagle's medium (DMEM), (2) DMEM with 50% AF, or (3) DMEM with 50% fetal calf serum (FCS). Transcriptional changes were analysed using microarray and enriched with WebGestalt and Enrichr. Additionally, eight diagnostic genes were analysed using semiquantitative real‐time PCR to investigate epidermal differentiation and cellular stress after HA exposure as an alternative for AF exposure. The AF and FCS treatments resulted in enrichment of genes relating to varied aspects of epidermal and keratinocyte biology. In particular, p63‐, AP1‐ and NFE2L2‐ (Nrf2) associated genes were found significantly regulated in both treatments. More genes regulated by FCS treatment were associated with inflammatory signalling, whilst AF treatment was dominantly associated with molecular establishment of epidermis and lipid metabolic activity. HA exposure mostly resulted in gene regulation that was congruent with the AF microarray group, with increased expression of ITGA6 and LOR. We conclude that AF exposure enhances keratinocyte differentiation in vitro, which suggests that AF constituents can be beneficial for wound‐healing applications.

## 1 INTRODUCTION

Cutaneous wound healing is a clinically important field of research where the translation of new treatment options is desperately needed. The healing process involves innate and adaptive immunity. Blood factors and all the cells and compartments of the integumentary system compromise a complex structure that presents many potential targets for intervention. Inflammatory cascades and the re‐establishment of epithelial integrity marks the early phase of cutaneous wound healing whilst tissue remodelling and the return of further skin functions occur at later stages of the healing process.^1^

The realisation that early fetal wounds heal with tissue regeneration instead of scarring suggests an instructive role of the embryonic environment in regulating the response to wounding.^2^ The human fetus is immersed in amniotic fluid (AF)^3^, ^4^ which is rich in epithelial growth factor (EGF), transforming growth factors alpha and beta 1 (TGFα, TGFβ1), insulin‐like growth factor 1 (IGF1), erythropoietin (EPO) and tissue factor, as well as several protective factors such as lactoferrin, α‐defensin, lysozyme, calprotectin and cathelicidin.^5^ These factors play important roles in adult wound healing.^6^ Hyaluronic acid (HA) is a major component in AF, and it also has higher concentration in younger than in older connective tissue.^7^ The presence of HA in fetal wound healing is prolonged compared to that of the adult.^2^ Exposure to AF shortens the time to re‐epithelialization of human adult wounds, and this effect is mediated by HA.^8^ Moreover, treatment of wounds with HA, in a minimally invasive in vivo human wound model, has been shown to accelerate re‐epithelialisation and alter protein expression.^9^

Several factors, including TGFβ1, EGF and HA, have seemed likely promising therapeutic candidates for wound regeneration. To date, their clinical impact has been moderate and it seems unlikely that single‐agent therapies are the key to solving the complex processes of repair and regeneration in human cutaneous wound healing.^10^

There is a need to further characterise the keratinocyte phenotype under AF stimulation by analysing gene expression changes from a broader perspective. Such cell culture experiments on human primary cells can bridge gaps between in vitro and in vivo experiments. Identifying transcriptional changes and their biological relevance provides a comparative baseline for changes occurring in a more complex tissue setting. Insights gleaned herein provide an enriched understanding of epidermal healing under AF exposure, which furthers the research into potential clinical use of AF and its constituents as candidates for wound‐healing therapy.

## 2 MATERIALS AND METHODS

### 2.1 Human primary cell cultures

All experiments involving human material were performed under ethical approval from the Swedish Ethical Review Authority (register number 2018/97‐31) and in accordance with ethical standards at the Linköping University and Swedish and European regulations. Skin was obtained from healthy female patients undergoing routine breast reduction surgery, and all material was de‐identified.

Briefly, human keratinocytes were isolated through mechanical dissection and incubated in 15 ml Dispase (Gibco, Life Technologies; 16.7 mg/ml, 1.04 U/ml) for 18 h at 8°C. The epidermis was transferred to 2 ml ethylenediaminetetraacetic acid (EDTA; 0.02%) and 2 ml trypsin (Invitrogen, Life Technologies) to dissociate cells. Cells were washed in Dulbecco's modified Eagle's medium (DMEM) with 10% fetal calf serum (FCS) and centrifuged at 400× *g* for 5 min. The cells were seeded in T‐75 culture flasks (Falcon) in complete keratinocyte serum‐free medium (KSFM; Invitrogen, Life Technologies) supplemented with 25 µg/ml bovine pituitary extract (BPE) and 0.2 ng/ml EGF as provided by the manufacturer, and 50 U/ml penicillin and 50 mg/ml streptomycin (Gibco BRL, Life Technologies).

### 2.2 Collection of amniotic fluid

Residual AF was obtained from ultrasound‐guided amniocenteses from women who were 14–18 weeks pregnant and gave informed consent. The collection was approved by the ethics committee at Linköping University, Sweden (register number 03–342). The AF from approximately 100 women were pooled and centrifuged at 2400× *g* for 5 min. The supernatant was filtered through a sterile 0.22 µm pore‐size filter (EMD Millipore), and aliquots of AF were kept at −20°C.

### 2.3 Cell culture treatments

At passage three (P3) subconfluence, six keratinocyte cultures in 75 cm^2^ cell culture flasks were starved (DMEM only) for 12 h and then randomised into three groups. Two cultures were subsequently given only DMEM, two were kept in DMEM with 50% AF and two received DMEM with 50% FCS. All cultures were trypsinised and pelleted 24 h later.

Cell counting was performed by staining an aliquot of the trypsinised cell suspension in Trypan Blue and quantifying total, live and dead cells in the EVE Automatic cell counter (NanoEnTek). Cell counts are given as live cells per ml and viability as % of total cells counted. Keratinocyte cultures in KSFM, DMEM and DMEM with 50% FCS (*n* = 6) were quantified for proliferation and viability at 24 and 48 h and presented as mean with SD (Figure S1).

### 2.4 Gene expression analysis with microarray

The duplicate cell pellets were pooled and sent into plastic tubes on dry ice (−70°C) to the core facility for Bioinformatics and Expression Analysis at Karolinska Institute (Stockholm, Sweden) for microarray processing. In brief, total RNA was isolated using the RNeasy kit (Qiagen), performed according to the manufacturer's instructions. The Affymetrix HG‐U133 plus 2.0 arrays with GeneChip One‐Cycle Target Labelling and Control Reagents, the Affymetrix Hybridization Oven 640 and GeneChip Fluidics Station 450, as well as the Affymetrix GeneChip Scanner 3000 were used in accordance with the manufacturer's recommended protocols. CEL files were retrieved through GCOS 1.4. Gene expression analysis was conducted using MAS5 to gain flags (P = present, A = absent) as well as RMA normalisation followed by the MvA analysis using CARMAWeb.^11^ CEL files and RMA data are available at GEO (GSE182704). Probeset expression was summed per gene, and lists were created showing top 100 upregulated and downregulated genes, as well as probesets showing 2‐fold increased expression and log2 signal above 3 through the MvA analysis. Venn diagrams were created using Venny 2.1 (https://bioinfogp.cnb.csic.es/tools/venny/), which spawned further gene lists and comparisons. Cluster mapping and PCA plotting were done through ClustVis (https://biit.cs.ut.ee/clustvis).^12^

We used WebGestalt (http://bioinfo.vanderbilt.edu/webgestalt)^13^ for GSEA of Gene Ontology terms, known pathways (KEGG, PathwayCommons, WikiPathway) and transcription factor targets. We used the following standard settings for WebGestalt GSEA: the hypergeometric statistical methods with Benjamini–Hochberg multiple testing adjustment and set hsapiens__affy_hg_u133_plus_2 as reference set for enrichment analysis. Minimum number of genes was 5 and FDR set to <0.05. We also used Enrichr (https://maayanlab.cloud/Enrichr/) to mine several databases and repositories for our gene sets to extrapolate biological relevance of gene expression changes. Unless otherwise noted, false discovery rate (FDR) or adjusted *p*‐values were used for ranking and significance thresholding. Figures summarising findings were constructed in Inkscape: Open‐Source Scalable Vector Graphics Editor v1.0.1 (The Inkscape Project; https://inkscape.org).

### 2.5 Quantitative real‐time PCR

Exploring the relationships between the starvation treatment (DMEM), HA and FCS treatment in terms of keratinocyte differentiation and activity, we selected eight standard markers for semiquantitative real‐time PCR (Figure 4C). The starting primary human keratinocyte culture (“Ctrl”) is used as reference (set to 1). The DMEM serum starvation treatment (“Starve”) is the same for the AF and FCS groups, but from 24 h and onwards, the treatments differ.

Primary keratinocytes were seeded at 100 000 cells/ml in 6‐well plates. Control keratinocytes were trypsinised, washed (in phosphate‐buffered saline (PBS)) and pelleted (by centrifugation at 200× *g* for 5 min), and the remaining samples were starved for 24 h in DMEM. After starvation, medium was changed to either DMEM with 0.1‐mg/ml HA (Sigma‐Aldrich, Hyaluronic Acid molecule weight 1.5–1.8 × 10^6^ Da) or DMEM with 50% FCS. Replicate samples were trypsinised and pelleted after 24, 48 and 72 h. RNA from each group was isolated using PureLink RNA Mini Kit (Thermo Scientific) according to manufacturer's protocol. Reverse transcription was carried out using High‐Capacity RNA‐to‐cDNA synthesis kit (Applied Biosystems) according to the manufacturer's protocol. Yield and purity were determined spectrophotometrically using NanoDrop 2000 (Thermo Scientific). Real‐time PCR using Fast Universal PCR MasterMix (Applied Biosystems) was carried out with ABI 7900HT Fast Real‐Time PCR system (Applied Biosystems) in 96‐well plates. Hydroxymethylbilane synthase (HMBS) was used as endogenous control, previously selected through the use of a TaqMan Express Human Endogenous Control Plate (Applied Biosystems). For primer assays used, see table S1 (TaqMan Gene Expression Assays 20X, Applied Biosystems). Relative quantification was performed using the ΔΔC(T)‐method, resulting in fold changes relative to control. Technical replicates were averaged for each biological replicate, and duplicate cultures were used for each group. Graphs were created in Prism 7.0 (GraphPad Software).

## 3 RESULTS

### 3.1 Microarray gene expression analysis

**FIGURE 1:**
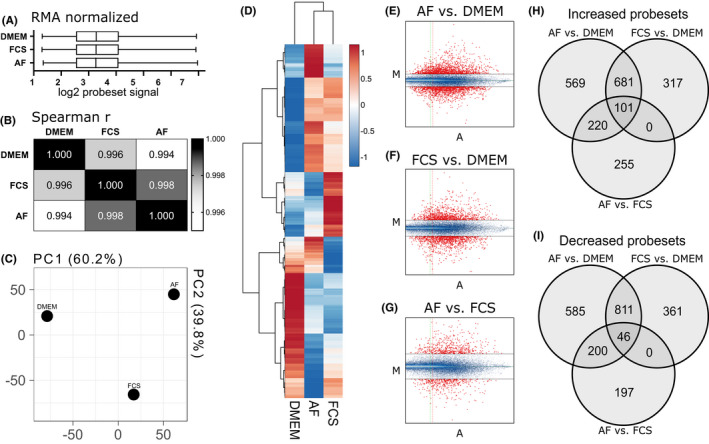
Overview of array experimental data. (A) RMA normalised log_2_ values across samples. (B) Spearman's correlation between groups. (C) Principal component analysis showing PC1 and PC2. (D) Heatmap with hierarchical clustering of top 1000 genes and all groups. (E‐G) MvA plots for each comparison: (E) amniotic fluid (AF) vs. DMEM, (F) FCS vs. DMEM and (G) AF vs. FCS—probesets above or below 2‐fold change are shown in red, and the threshold of A = 3 is shown as a vertical line; (H) Venn diagram showing probesets that are marked as “Increased”, meaning upregulated, in all the three comparisons. (I) Venn diagram showing “Decreased” probesets. A = ½ (log2 (experiment ×reference); AF, amniotic fluid–exposed cultures; DMEM, serum‐starved cultures; FCS, fetal calf serum–exposed cultures; M = log_2_ (experiment/reference); PC, principal component; RMA, robust multiarray averaging

We performed basic quality control on the arrays, observing normalised signal distribution after RMA (Figure 1A) that is highly correlated (Figure 1B). Principal component analysis (PCA; Figure 1C) indicates that starvation had the greatest impact on variation (interpreted as PC1), whilst the difference between AF and FCS explained the rest of the variation in the data set (interpreted as PC2). When looking at the top 1000 expressed genes, the group clustering follows PCA results with FCS and AF groups slightly closer to each other than the DMEM group (Figure 1D). The MvA plots for each comparison, AF versus DMEM (Figure 1E), FCS versus DMEM (Figure 1F) and AF versus FCS (Figure 1G), show y‐axis (“M”) representing fold change (log2) and x‐axis (“A”) representing relative abundance. The overview of probeset‐level expression changes is presented in Figure 1H (“increased”) and Figure 1I (“decreased”). There were 1571 probesets increased in AF treatment compared with DMEM and 1642 decreased. They represent 813 upregulated and 1099 downregulated genes. The FCS rescue of starvation resulted in increased signal in 1099 probesets, representing 621 genes, and decreased signal in 1218 probesets, representing 785 genes. The third comparison, between AF rescue and FCS rescue of starvation (represented by PC2 in Figure 1C), resulted in 576 probesets increased (Figure 1H) and 443 decreased (Figure 1I), representing 366 and 309 genes, respectively. The RMA normalised probeset data are available in a supplementary file (.xlsx‐format) and at GEO: GSE182704.

**FIGURE 2:**
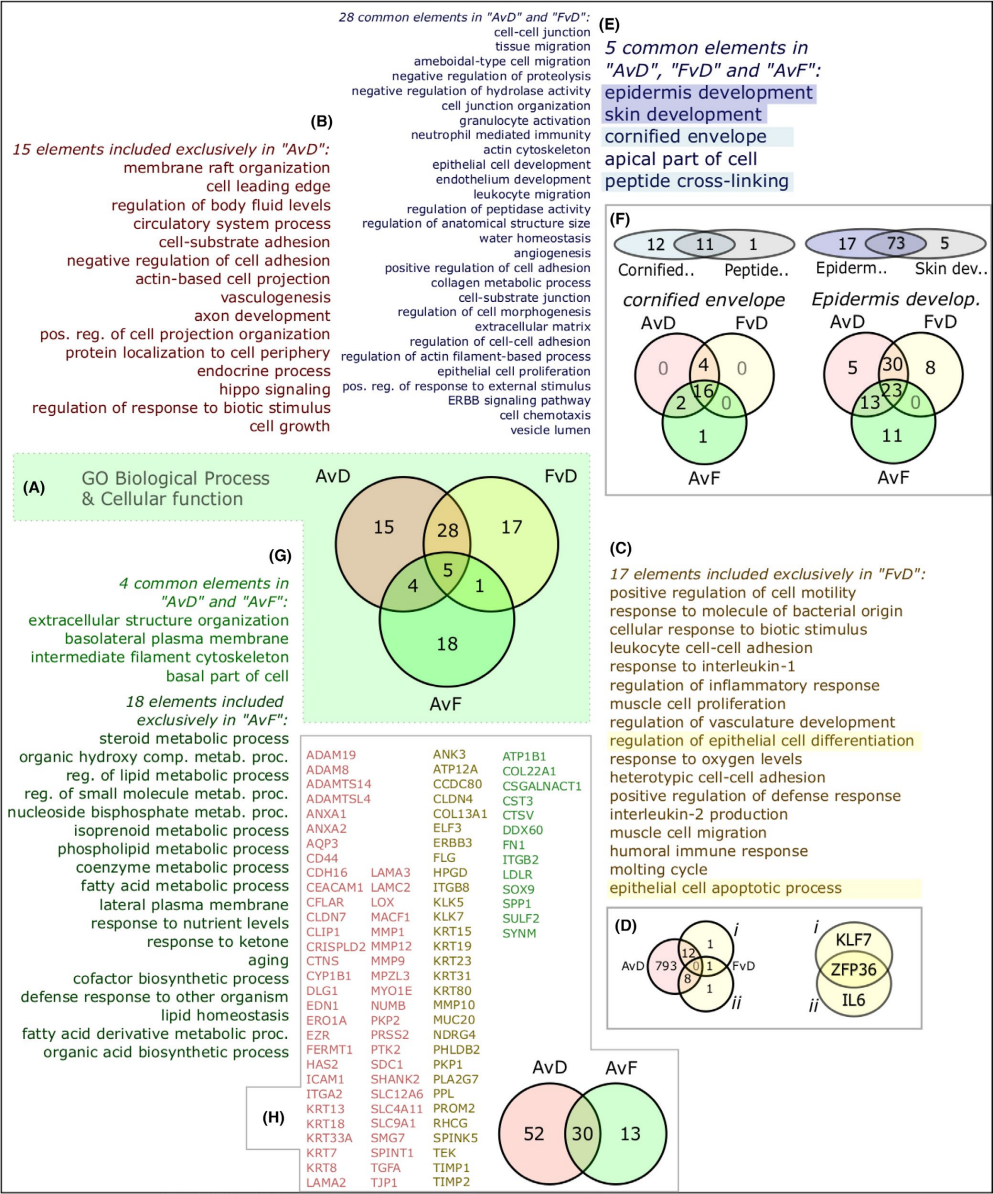
Gene Ontology (GO) gene set differential analysis and interpretation. (A) Significant GO terms among upregulated genes shown as sets for all three comparisons. (B) Unique terms ^15^ for amniotic fluid (AF) treatment vs DMEM group (AvD). (C) Unique terms ^17^ for fetal calf serum (FCS) treatment vs DMEM (FvD). (D) Genes representing epithelial‐related terms, (i) “regulation of epithelial cell differentiation”; (ii) “epithelial cell apoptotic process”). (E) Enriched GO terms in AF and FCS groups compared with DMEM (28 and 5, respectively). (F) Sets of genes related to terms “cornified envelope” and “epidermis development”. (G) Enriched terms in the AF versus FCS comparison (4 and 18 terms, respectively) of which 18 were not also increased in the AvD comparison. (H) Expansion of the four terms in AvD intersecting AvF into gene sets shows 13 genes (in green) that signify AF over FCS treatment and 52 (in red) that are increased in AF vs. DMEM, with an overlap of 30 (yellowish) satisfying both criteria. AvD, AF vs. DMEM; FvD, FCS vs. DMEM; AvF, AF vs. FCS

These lists of genes, separated into upregulated and downregulated (increased/decreased), were individually run through Enrichr and WebGestalt tools. Gene Ontology (GO) terms for upregulated subsets of genes are summarised in Figure 2. Central to this analysis is the discovery of GO terms enriched among sets of (increased) genes in the tripartite comparison: AF versus DMEM (AvD), FCS versus DMEM (FvD) and AF versus FCS (AvF) (Figure 2A). Identifying commonalities and differences sheds some light on the biological relevance of AF treatment.

The main themes among the set of 15 terms associated with the AF treatment (Figure 2B) relate to regulation of cell adhesive properties, cell growth and the hippo pathway. The subset exclusive to FCS treatment shows 17 exclusive GO terms (Figure 2C) related to innate and immune regulatory activity, proliferation and migration. There are also terms specifically related to epithelial cells (“regulation of epithelial cell differentiation” and “epithelial cell apoptotic process”). We compared the genes associated with these two terms to the 813 upregulated genes in AF treatment (Figure 2D). Contrary to a view of set exclusivity, only three genes of 23 were unique to FCS treatment. These were KLF7 (Figure 2D‐i), IL6 (Figure 2D‐ii) and ZFP36.

There is a predominance of epidermis‐related functions among the shared terms of AF and FCS treatments (Figure 2E). Several terms relate to the microarchitecture of the epidermis, such as the cell–cell adhesion establishment and regulation of suprabasal layers, migration and remodelling of the basal layer keratinocytes and their adhesion to the substrate. Other terms relate to more functional annotation including water homeostasis, immunological signalling activity, ECM remodelling and polarity. Both the central set of 5 terms and the set of 28 terms are shared by AF and FCS treatments, but the central 5 terms are additionally represented by genes even higher under AF compared with FCS treatment. Analysis of term similarity shows that “epidermis development” and “skin development” are highly related, as are “cornified envelope” and “peptide cross‐linking” (Figure 2F). The distribution of genes related to the bigger of each pair of terms is represented in Venn diagrams. This shows a substantial similarity between the AvD and FvD comparisons but also distinct regulation. These genes relate to epidermis development, particularly late stages such as appearance of the cornified envelope.

We further unpacked both the set of 18 AvD‐exclusive terms as well as the set of 4 shared by the AvD and AvF comparisons (Figure 2G). The smaller set is dominated by polarity and structural organisation, congruent with interpretations of tissue architecture activity from Figure 2B. The 18 terms representing genes regulated beyond the FCS treatment are dominated by metabolic processes particularly relating to lipids. This is congruent with interpretations of late epidermal development upon AF treatment (Figure 2F). The genes representing the smaller set of 4 shared terms are combined and represented in a Venn diagram (Figure 2H), with 30 shared genes between the AvD and AvF comparisons, and 52 and 13 exclusive genes, respectively. Among them are keratins, ECM‐regulating genes and transcriptional regulators specifically relevant to keratinocyte biology.

**FIGURE 3:**
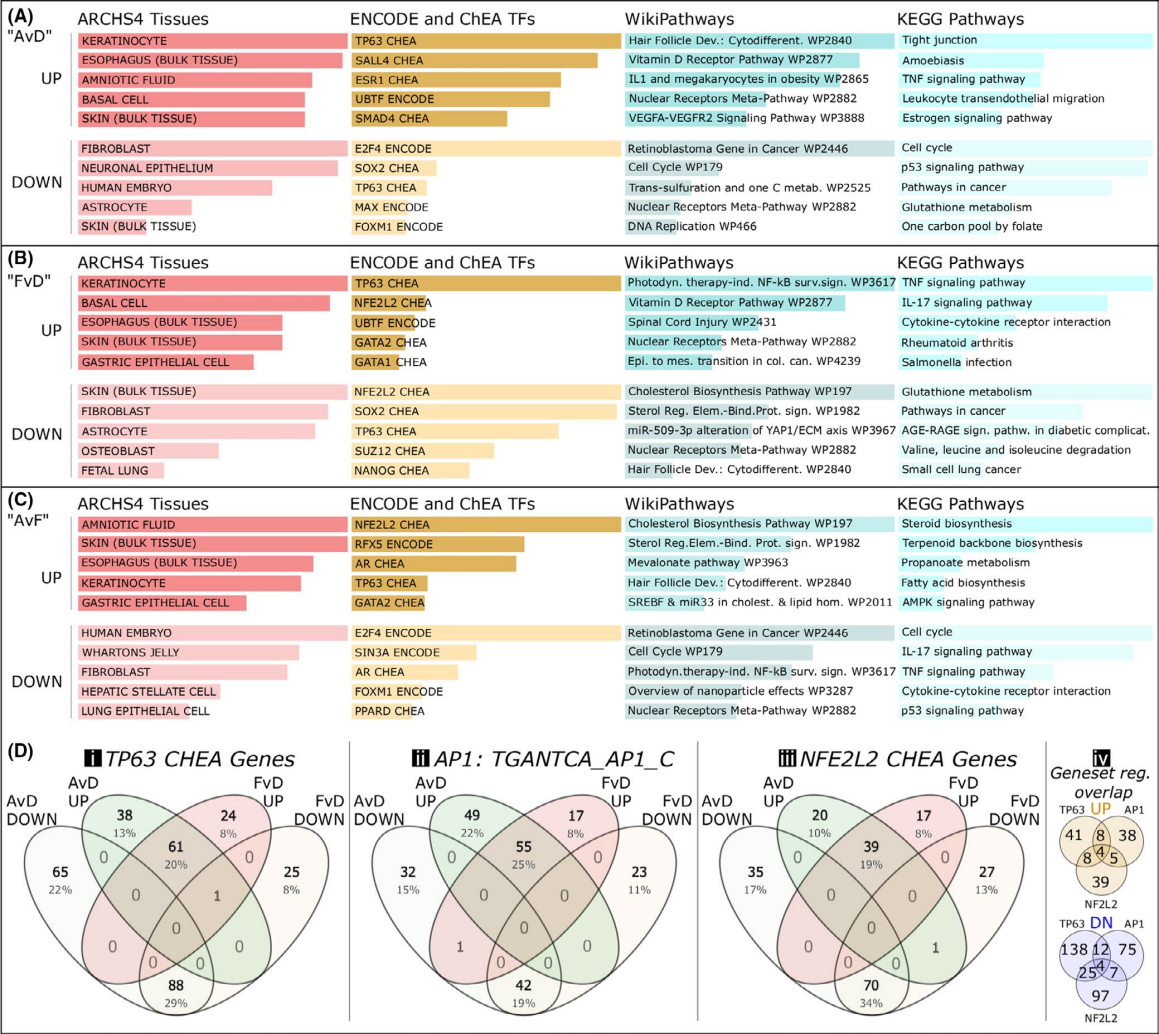
Categories of genes regulated “up” or “down” (>2‐fold change) in the three comparisons, with examples of major keratinocyte transcription factor network associations. (A) Amniotic fluid rescue of starvation (AF vs. DMEM; “AvD”). (B) Fetal calf serum rescue of starvation (FCS vs. DMEM; “FvD”). (C) Amniotic fluid versus fetal calf serum regulatory changes (AF vs. FCS; “AvF”). (D) Three major transcription factor networks and their genes as apparent in the >2‐fold regulation, presented in 4‐way Venn diagrams: (i) TP63 (tumor protein p63), as selected by ChIP data ChEA; (ii) AP1 (activator protein 1 complex), gene set by binding sequence TGANTCA according to mSigDB; (iii) NFE2L2 (a.k.a Nrf2), as selected by ChEA data through Enrichr (www.enrich.net); (iv) showing overlaps of the gene set collections in (i‐iii), divided into upregulation and downregulation, indicating network module set similarities in our data. Bars represent combined *p*‐value and rank scoring, and all are considered significantly enriched

When using the publicly available database “ARCHS4 tissues”, both AF (Figure 3A) and FCS (Figure 3B) treatments show clear relevance to the epidermis with terms such as “Keratinocytes”, “Basal cell” and “Skin (Bulk tissue)”. The AF treatment additionally showed high ranking of “Amniotic fluid” over FCS (Figure 3C). Among the transcription factor network associations, as presented through ENCODE and ChEA data sets, TP63 appears relevant in all comparisons (Figure 3A‐C). For the AF treatment (Figure 3A), we also see SALL4, ESR1, UBTF and SMAD4 among the top five upregulated associations, whilst E2F4, SOX2, MAX and FOXM1 figurate at the top of the downregulated associations. Similar regulation is found in the FCS group (Figure 3B), although NFE2L2, UBTF and GATA1/2 figurate among the top five terms whilst others are found further down the list. For decreased genes, the top associations are SOX2, as with AF treatment, as well as SUZ12, NANOG and NFE2L2. In the AF versus FCS comparison (Figure 3C), the top terms also include NFE2L2, RFX5 and TP63.

Among the top five pathway terms in both treatments, we find “Vitamin D receptor pathway”, “Nuclear receptor meta‐pathway” and “TNF signalling pathway.” The term “Tight junction” was the highest ranked KEGG pathway in AF treatment but did not make it into top five terms in FCS treatment. Instead, higher rankings were seen in FCS treatment for “IL‐17 signaling pathway” and “Photodynamic therapy‐induced NF‐kB survival signaling”. Analysis of the genes differentially regulated between the treatment groups (AvF; Figure 3C) leads to similar themes as the GO analysis, in Figure 2G. The top four KEGG terms clearly relate to lipid metabolism, whereas “AMPK signaling” further underscores increased cellular homeostatic activity. The IL‐17, TNF and NF‐kB activities are higher after FCS treatment than after AF treatment.

The relevance of the p63 (TP63) network for epidermal development and keratinocyte differentiation motivates an analysis of the representation of this network among the genes in this study. Also important for keratinocytes is the AP1 transcriptional network. Apparent through our analyses, the NFE2L2 (Nrf2) network is also of some significance to the keratinocyte response to the two treatments. We therefore constructed Venn diagrams (Figure 3D) of TP63‐related genes (Figure 3D‐i), AP1‐related genes (Figure 3D‐ii) and NFE2L2 genes (Figure 3D‐iii) to see some similarities and differences in gene representation.

Analysis of TP63 differences (Figure 3D‐i) showed 252 significantly regulated genes in AF and 198 in FCS related to the TP63 ChEA database term compared with DMEM. A slight majority of genes are shared between both treatments in both regulated directions (increased and decreased), but there are more distinct genes in the AF condition. Segregating gene sets into Venn diagrams together with published data on clustered p63 binding sites and their adjacent genes,^14^ we found 61 shared genes in the AF and FCS treatment, 59 genes exclusive to the AF treatment, and 17 exclusives to the FCS treatment (not shown). In the AvF comparison, 33 additional p63‐related genes are exclusively upregulated. Another recursive enrichment analysis was used to search for known p63 co‐transcription factors to better fingerprint the differences between the two treatment groups. Some top‐ranked results explaining the exclusive set of upregulated genes in the AF treatment include GRHL1, TEAD1, ZNF532, ZNF217, KLF5, LEF1, CTNNB1 and MYC. The exclusive set of 17 genes associated with the FCS treatment is considered small but resulted in enrichment for networks associated with PIAS1, UBF1/2, E2F6, RELA, NFKB1, STAT3, ZNF697 and ARNTL2. The shared set of 61 genes between AF and FCS treatments were associated with JUNB, IRF8, STAT5A, CEBPB, JUN, TCF4, EHF, IRF6, CTNNB1, POU2F3 and FOXN1. The 33 genes exclusive to the AvF direct treatment comparison were associated with RFX5, CJUN, JUN, WT1, ZNF217, VDR, RORA, TFAP2C, ZNF750, ZNF608, ZNF117, FOXA2, SMAD3 and SIN3A/B. Altogether, distinct p63 co‐transcription factors can explain some of the differences between p63 network activity under the different treatments.

AP1 is a transcription factor complex consisting of c‐Fos and c‐Jun^15^ and is a key regulator of epidermal homeostasis, the EDC architecture and keratinocyte activity^16^ (Figure 3D‐ii). Gene regulatory activity shows that the bulk of AP1‐associated genes are shared in both upregulation and downregulation between the treatments (Figure 3D‐ii). Again, there is a numerical advantage to the AF treatment in AP1 representation in both upregulated and downregulated sets. In fact, about half of the AP1‐associated upregulated genes in AF are shared with FCS treatment, whilst more than 75% of AP1‐associated genes upregulated by FCS are shared with AF. Unlike the p63‐associated sets, the AP1‐associated genes are more numerous among the upregulated rather than downregulated gene sets indicating an increase in AP1 activity overall.

NFE2L2 (Figure 3D‐iii), also commonly known as Nrf2,^17^ represents an important network related to stress, metabolism and wound‐healing responses of keratinocytes.^18^, ^19^, ^20^ NFE2L2 is represented in both treatment groups. The associated genes are more evenly distributed between treatments, but also more highly represented among the downregulated genes. The genes MAFF and MAF are regulated in both treatments, encoding for heterodimer binding partners to Nrf2.^21^ Identified Nrf2 target genes, such as GCLM, SLC6A6 and PIR,^22^ decrease in both treatments.

Overlaps between all the genes in Figure 3Di‐iii are constructed in Figure 3D‐iv to check confirm that this analysis does not rely on fully overlapping gene sets. In the upregulated Venn diagram, the 4 genes in the middle are EHF, LAMC2, CST6 and SCEL, and the 8 shared between TP63 and AP1 are IL1RN, ELK3, KRT8, DSC2, ANKRD22, PITPNC1, ELOVL4 and HBEGF. The 8 genes shared between TP63 and NFE2L2 are CYP1B1, LMO7, SLC44A3, DUSP4, ANXA1, PLAUR, PPARD and QPCT. The 5 genes shared between NFE2L2 and AP1 are PCDH9, NPEPPS, MACF1, CXCL5 and CLDN4.

**FIGURE 4:**
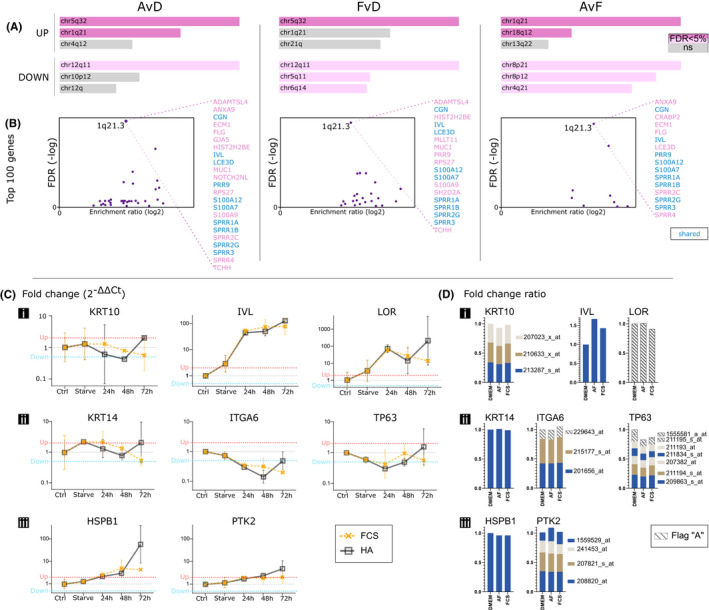
Chromosomal differentiation cluster analyses and qPCR with hyaluronic acid (HA) treatment to substitute for amniotic fluid (AF). (A) Chromosomal location enrichment among upregulated and downregulated genes per comparison. (B) Enrichment of the epidermal differentiation complex (EDC) at location 1q21.3 among Top‐100 increased genes in all treatments, with specified gene lists. (C) qPCR measured fold changes for three suprabasal, three basal and two stress genes after substituting AF with HA. (D) Visualisation of probesets corresponding to the selected genes to compare gene expression between qPCR and microarray methods and HA/AF treatments. Flag A = absent, AF, amniotic fluid; Ctrl, human primary keratinocytes; FCS, fetal calf serum; HA, hyaluronic acid

The importance of certain chromosomal locations for differentiation of keratinocytes motivated an analysis of chromosomal location enrichment. The top three ranked chromosomal locations, using Enrichr, include chr1q21 (a definition that includes the EDC but may be broader) among the upregulated genes in each comparison (Figure 4A). Using WebGestalt, the enrichment scores for the EDC are even higher among the top 100 increased genes in each comparison (Figure 4B). The shared genes in all comparisons at the EDC cluster are shown in blue.

### 3.2 Quantitative real‐time PCR

Figure 4C‐i shows suprabasal differentiation markers. They are mostly upregulated over time, except KRT10, which shows initial decrease, then recovery in the HA group and a delayed decrease in the FCS group that does not recover within 72 h. The second row of genes in Figure 4C‐ii represent three basal keratinocyte markers. As with KRT10, the KRT14 response is an initial decrease followed by a sharp increase within 72 h in the HA group, whilst the FCS treatment results in a delayed decrease. There is a relative increase of six of the eight genes after 24‐h starvation. Only the basal ITGA6 (Integrin alpha 6) and TP63 decrease after starvation treatment, a decrease that continues across the first 24 h of HA and FCS treatments. The expression of ITGA6 remains relatively stable in the FCS group after the initial decrease, but there is a sharp rise in the HA group after 48 h. The two genes in Figure 4C‐iii, HSPB1 (Heat Shock Protein Family B (Small) Member 1) and PTK2 (Protein Tyrosine Kinase 2), are used as markers of cellular stress activation. Their expression rises steadily over time, except at the 72‐h timepoint for HSPB1 where the HA group shows a sharp increase. At 72 h, the FCS group shows a levelling off for both genes, with HSPB1 and PTK2 remaining upregulated at levels similar to those at 48 h.

A summary of normalised and relative expression of these markers in the microarray data is shown in Figure 4D, with values from RMA and flags from MAS5. The transcript value in the DMEM group is set to 1 or the sum of all probeset values where there are several. For KRT10, the results are weaker but similar with a steady level for FCS 24 h after starvation and a slight decrease in AF. The differentiation marker IVL shows increases in both groups, slightly more in AF than FCS. This is directionally similar to the qPCR, but with lower differences in expression compared with DMEM and showing a difference between AF and FCS. The low expression of LOR in the microarray, and the Absent flag, does not match the qPCR results. For both methods and all treatments, the suprabasal markers show varied expression regulation, but the AF and HA treatments are directionally congruent for KRT10 and IVL. The basal markers show little change in the array data (Figure 4D‐ii), with KRT14 and ITGA6 remaining at starvation levels, unlike in the qPCR where ITGA6 markedly decreases. As in the qPCR data, KRT14 remains stable for the first 24 h of treatment, whilst TP63 shows an initial decrease in all treatments for the same period. The gene HSPB1 shows a slight decrease in the array data (Figure 4D‐iii), unlike in the qPCR data, and PTK2 is directionally more congruent with slight increase after AF treatment.

## 4 DISCUSSION

The results of our gene set enrichment analyses show two important aspects of AF treatment of primary human keratinocytes in the short term. The first is that the poststarvation rescue using AF is comparable with FCS in terms of re‐establishing an expanding culture with stratification potential. We previously found similar effects, at 50% concentrations, on the re‐epithelialisation of in vitro human skin wounds,^8^ showing some coherence between the results on epidermal regeneration and these keratinocyte culture data. The second is that there are differences in some of the core network‐associated transcriptional activities within the phenotypic space of keratinocytes attributable to AF. Through our use of multiple methods of analysis and multiple databases, we can catch several central biological interpretations as well as some more subtle phenotype attributes. The use of multiple databases for interpreting biological relevance can be more robust than relying on any single annotated source.^23^

Transcriptional changes in keratinocyte cultures are in many ways related to what can be expected in a wound‐healing scenario because many receptors and responses are keratinocyte specific. Our analyses show that there is substantial similarity between the AF and FCS treatments, some of which is attributable to the initial starvation condition. Serum starvation is a well‐used technique in cell culture experiments, although it is variably implemented and defined.^24^ Typically, starvation involves DMEM with no or low amounts of serum. KSFM contains more amino acids and hormones (more akin to Ham's F12) than the very basal DMEM formulation and was designed with enough supplements to typically allow serum‐free in vitro cultures. In this case, we chose to implement DMEM‐based starvation instead of KSFM without EGF and BPE to better reproduce previous uses of starvation paradigms.

Starvation is not unequivocally related to metabolic inactivity.^24^ Growing cells, such as in cultures that have not yet reached confluency, require higher levels of glucose owing to cellular anabolic activity and rRNA activity due to increased translation. The increased turnover of rRNA is helped by the UBTF‐dependent transcriptional promotion of rRNA genes,^25^ which we see enriched for in both AF and FCS poststarvation treatments. The enrichment of terms related to the NFE2L2 network also indicates an increase in cellular stress activity. NRFs can activate target genes termed antioxidant response elements, are crucial for mediating cellular stress responses and may be important for chemical and toxic protection in keratinocytes.^26^ The NFE2L2/Nrf2 is also a KGF target that regulates gene expression and inflammation in wound healing.^18^

Calcium concentration is a major determinant for keratinocyte differentiation.^27^ The basic expansion medium of KSFM contains low amounts of calcium to minimise differentiation and maintain a proliferative phenotype. We did not measure or control calcium levels in this study, but DMEM, AF and FCS are all expected to contain higher levels of calcium, which likely drives an initial stimulus towards differentiation. According to manufacturer, DMEM contains 1.8‐mmol calcium chloride and FCS may contain around 3.5–4 mmol,^28^ whilst AF contains around 2 mmol (2–2.5 mEq^29^). These levels are above recommended concentration for maintaining keratinocytes undifferentiated, which may accentuate the transcriptional activity around loci related to keratinocyte differentiation and epidermal development, such as chr1q21.3, and the p63 and AP1 networks.

We identified the p63‐extended transcriptional network as a central regulatory theme. This can be expected, as we are dealing with keratinocyte cultures, and p63 is considered a key transcription factor for the epithelial lineages, including keratinocytes.^30^, ^31^ Simultaneously, we saw that the two treatments regulate different players within the p63 network, despite their significant gene set overlap and shared net directional change. The keratinocyte phenotype is heavily reliant on several clusters of genes, all regulated downstream of p63 and co‐factors. The epithelial differentiation complex (EDC) located on chr1q21.3 contains genes for S100A1‐A13, involucrin, loricrin, late cornified envelope (LCE) genes and other important keratinocyte genes.^32^ We find the EDC highly enriched in our gene sets in all comparisons (Figure 4A). There are three more documented clusters of relevance to keratinocytes: the keratin type I loci at chr17q12‐q21 containing the acidic keratins (KRT9‐20),^33^, ^34^ the type II loci at chr12q11‐q14 containing the basic (type II) keratins (including KRT1‐8)^34^ and a 40 kbp locus on chr19.^35^ These were represented among the transcriptional changes in all groups, but not highly enriched. To understand processes in both physiological and pathological states, we need to systematically explore and understand the involvement of chromatin remodelers at discrete stages of epidermal differentiation.^36^ In our data, circumspect regulation is found for the involvement of genes such as PBX1, a known epigenetic regulator of the LCE subcluster of the EDC,^37^ in contributing to differential regulation between AF and FCS treatment.

We looked for evidence on the balance between proliferation and differentiation and whether this was differentially regulated. Firstly, the GO terms, pathways and transcription factor results did not fit into such an interpretation between AF and FCS treatments, whilst there seemed to be clear differences in cell growth, metabolism and differentiation compared to DMEM (starvation). Differences in p63 and AP1 networks are not easily interpreted in this light, either. Whilst there is a slight advantage to the AF treatment in the number of genes associated with the subset of postulated progenitor‐related p63‐associated genes^38^ (50 in AF vs. 23 in FCS, besides 67 shared), it is not clear that the two treatments result in a difference of the categorical distribution of keratinocytes along the proliferation differentiation axis.

However, two major themes emerged from the AF analysis leading us to postulate a higher quality of epidermal development than under FCS. Lipid and sterol metabolic processes, as evidenced from pathway and GO analyses, correlated with late differentiation markers (related to cornified envelope) to hint at a more developed late differentiation repertoire among the differentiating subset of keratinocytes. This may indicate that although the proportions of differentiating cells are similar under both FCS and AF treatments, the transcriptional landscape under AF stimulus is more permissive for complete differentiation even in vitro. The second theme relates to increased activity around cell polarity, extracellular organisation and cell–cell interactions, something we consider important for the tissue microarchitecture, and particularly the basal niche. The AF treatment provides additional cellular spatial cues over those shared with the FCS treatment.

Both themes can be related to increased AP1 network activity in AF‐treated cells. Unlike the p63‐associated sets, the AP1‐associated genes are more numerous among the upregulated rather than downregulated genes indicating an increase in AP1 activity overall. This may be congruent with a view of AF being more supportive of in vitro epidermal development and keratinocyte differentiation, as suggested by microarchitectural attributes such as cell adhesion, polarity, hippo signalling and lipid metabolism. The dominance of “Tight junction” and “extracellular structure organisation” exemplifies that cell–cell interactions and the development of the epidermal microarchitecture are stronger in the AF treatment group. This may also be congruent with the appearance in the enrichment analysis of YAP activity and the hippo pathway. The hippo pathway, which is part of the developmental processes controlling organ size,^39^ could be a bridge between the two themes of adhesion and cell growth indicating a predominant theme of developmental tissue architecture. This is interesting from the perspective of the sourcing of the amniotic fluid—clearly related to early development—and the organismal boundary role^40^ of keratinocytes and epithelia. The hippo pathway is involved in skin wound healing through YAP/TAZ activity regulated by integrin binding,^41^ and YAP1 activity downstream of 14–3–3 regulation controls keratinocyte proliferation.^42^ YAP1/TAZ‐TEAD networks are intertwined with KLF4, potentially linking the repressive role of KLF4 for stem cell maintenance, and its role to promote differentiation, with extracellular chemical and mechanical cues.^43^

The underlying mechanisms behind the previously shown positive effects of AF on adult cutaneous wound healing^9^ might be better understood by a closer look at the genes regulated in cell culture. As an example, we found enrichment of the SALL4 transcriptional network, which is implicated in scar‐free healing through a role in ECM remodelling.^44^ The AF may be triggering keratinocyte differentiation that includes a higher degree of regeneration. Further studies are required to determine which factors are the crucial drivers, but a central role for HA has already been determined.^8^, ^9^ In our initial comparisons of HA and AF stimulus, we observe similar regulation and distribution of keratinocyte differentiation.

We conclude that the vast networks that govern epidermal homeostasis and the keratinocyte phenotypes show a metastability in regulation that allows both AF and FCS treatments to regulate similar functions through diverse gene set regulation. AF seems to drive maturation and regeneration in favour of inflammation in this experimental model, and the phenotype distribution in culture seems to be maintained also with HA. The analysis of gene expression changes may clarify future experiments in more complex models.

## CONFLICT OF INTERESTS

The authors declare that there is no conflict of interest regarding the publication of this paper.

## AUTHOR CONTRIBUTIONS

EN and GK planned the study; EN collected the AF, performed cell cultures for the microarray analyses and wrote the first draft with help from GK. EL, JR and JJ performed cell cultures and following PCR analyses. JR and JJ performed gene expression analyses. All authors analysed and interpreted the data and approved the final version of the manuscript.

## Supporting information

Figure S1. Photographs taken at 20X (200X) of keratinocyte cultures after 48 h in (A) KSFM, (B) DMEM only and (C) DMEM with 50% FCS. (D) Quantification of cells per ml at seeding (time 0 h), after 24 h and after 48 h for each medium group, with box and whisker plot of mean, min and max. (E) Bar charts of mean with SD of % viability of counted cells in each group, at each time. Scale bar for A‐C is 100 µM. (D, E: *n* = 6).

Table S1. Table of TaqMan assays and gene targets in qRT‐PCR.

## Data Availability

Microarray data is available at the NCBI Gene Expression Omnibus, accession number GSE182704: https://www.ncbi.nlm.nih.gov/geo/query/acc.cgi?acc=GSE182704.
