# Supplementary figures and images for: Effects of amniotic fluid on human keratinocyte gene expression: Implications for wound healing
Source: Exp Dermatol. 2022 Jan 15;31(5):764–74. doi: 10.1111/exd.14515 (PMC9305168; doi:10.1111/exd.14515)

**S1**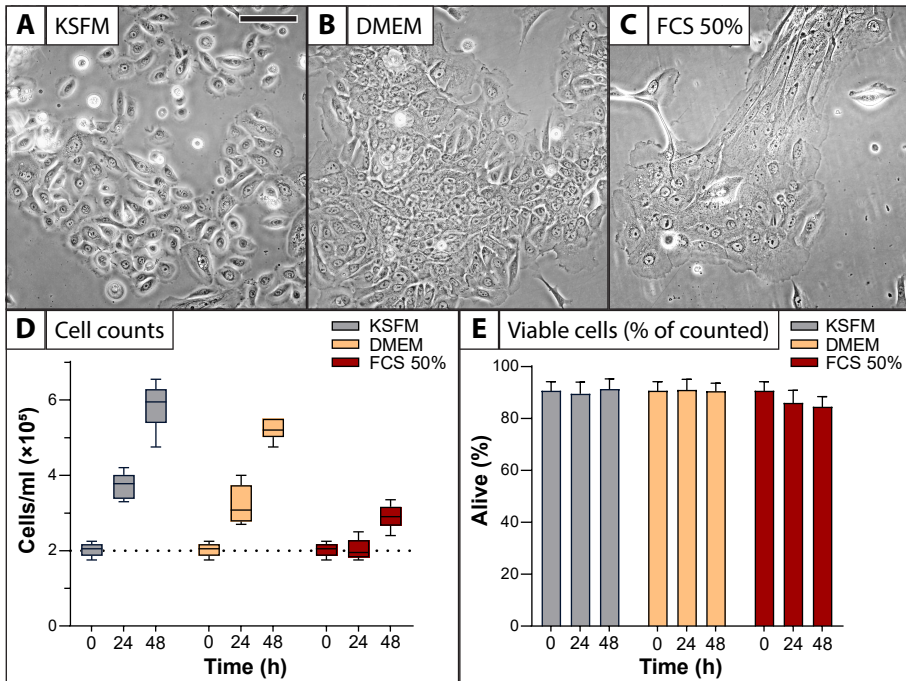

Supplement: Supplementary file 1 — Figure S1. Photographs taken at 20X (200X) of keratinocyte cultures after 48 h in (A) KSFM, (B) DMEM only and (C) DMEM with 50% FCS. (D) Quantification of cells per ml at seeding (time 0 h), after 24 h and after 48 h for each medium group, with box and whisker plot of mean, min and max. (E) Bar charts of mean with SD of % viability of counted cells in each group, at each time. Scale bar for A‐C is 100 µM. (D, E: n = 6). [file EXD-31-764-s002.pdf]
