# Supplementary material for: Effects of amniotic fluid on human keratinocyte gene expression: Implications for wound healing
Source: Exp Dermatol. 2022 Jan 15;31(5):764–74. doi: 10.1111/exd.14515 (PMC9305168; doi:10.1111/exd.14515)
Supplement: Supplementary file 2 — Table S1. Table of TaqMan assays and gene targets in qRT‐PCR. [file EXD-31-764-s001.docx]

**Tables**

**Supplementary Table 1**

| **Protein** | **Gene** | **Identity** |
| --- | --- | --- |
| Keratin 10 | KRT10 | Hs00166289_m1 |
| Involucrin | IVL | Hs00902520_m1 |
| Loricrin | LOR | Hs01894962_s1 |
| Keratin 14 | KRT14 | Hs00265033_m1 |
| Integrin Alpha-6 | ITGA6 | Hs01041011_m1 |
| Tumor Protein P63 | TP63 | Hs00978340_m1 |
| Heat Shock Protein Family B Member 1 | HSPB1 | Hs00356629_g1 |
| Protein Tyrosine Kinase 2 | PTK2 | Hs01056457_m1 |
